# Workplace injury and associated factors among construction workers in Gondar town, Northwest Ethiopia

**DOI:** 10.1186/s12891-019-2917-1

**Published:** 2019-11-09

**Authors:** Fentahun Berhanu, Mulat Gebrehiwot, Zemichael Gizaw

**Affiliations:** 1 Lideta sub-city administration, Gondar, Ethiopia; 2 Department of Environmental and Occupational Health and Safety, Institute of Public Health, College of Medicine and Health Sciences, University of Gondar, Gondar, Ethiopia

**Keywords:** Occupational injury, Construction industry, Northwest Ethiopia

## Abstract

**Background:**

The construction industry is one of the most hazardous working areas, where the highest number of labourers engaged. However, the predisposing factors for occupational injury in the construction sites in Ethiopia are not well investigated. This study was, therefore, conducted to assess the magnitude of occupational injury and associated factors among construction workers in Gondar town, northwest Ethiopia.

**Methods:**

This cross-sectional study was conducted on 566 construction workers. Systematic random sampling technique was used to select study subjects. Data were collected using structured questionnaire and observation checklist. Multivariable binary logistic regression analysis was used to identify variables significantly associated with occupational injury on the basis of adjusted odds ratio (AOR) with 95% confidence interval (CI) and *p* < 0.05.

**Results:**

The overall prevalence of work-related injury in 3 months prior to the survey was found to be 39% (95% CI = 35.0–43.1%). The occurrence of occupational injury was associated with single workers [AOR = 0.50, 95% CI = 0.25, 0.97], longer service year [AOR = 2.79, 95% CI = 1.72–4.53], poor attention to work [AOR = 2.65, 95% CI = 1.33, 5.29], working with vibrating hand tools [AOR = 3.23, 95% CI = 1.19, 8.76], no aware about occupational hazards [AOR = 4.66, 95% CI = 1.99, 10.87], and alcohol consumption [AOR = 3.16, 95% CI = 2.09, 4.79].

**Conclusion:**

High prevalence of occupational injury was reported in the study area. Cut and fall were the leading causes. Marital status, service year, attention to work, use of vibrating hand tools, awareness about occupational hazards, and drinking alcohol were identified as factors associated with occupational injury. Therefore, health and safety trainings have to be taken place to aware workers about occupational injury and safety issues. Regular workplace supervision and provision of appropriate personal protective equipment (PPE) are also needed to prevent occupational injury. The findings of this study are useful to design and implement injury prevention strategies in the country. The study also contributes to the current literature as health and safety information is limited, especially in developing countries.

## Background

The construction industry has been considered an accident-prone industry. That is because construction sites are often filled with potential hazards that can lead to serious injury or death [1, 2]. Construction workers face a risk of fatal and non-fatal injury higher than any other groups of workers [3]. Although countries differ substantially in their structural industrial distribution or level of occupational health and safety, injuries in the construction industries are the major health and safety concerns in many countries [4]. Moreover, the rate of death of workers is higher in the construction industry than in any other industry [1].

Within the construction industry, the risk of fatality is 5 times higher than in manufacturing, whilst the risk of a major injury is 2.5 times higher [5]. The problem of death and injury as a result of the workplace accident has now been recognized as a global phenomenon. The construction industry accounts for 55,000 fatal injuries each year [1]. Besides, construction injury causes huge economic losses. Globally, the direct and indirect costs of fatal and nonfatal construction injury have been estimated over 10 billion USD per year [6].

Accident causalities in the construction industry is complex and multifaceted and accident prevention begins with having a clear understanding of those factors [7]. Personal and work characteristics combined with the working environment and organizational factors are believed toinfluence the creation of a hazardous environment that could be triggered by differentmechanisms that cause an accident [8–10]. Most of the accidents in the construction industry occurred because of insufficient safety measures [11]. Human errors are mainly responsible forconstruction accidents [12–14]. Site conditions or work environments play an important role in construction accidents [10]. Moreover, the temporary and transitory nature of construction sites are claimed to contribute to accidents [15]. While environmental factors such as climate, temperature, and geographical conditions could be considered as typical characteristics for construction sites [16]. Organizational factors may include characteristics referring to construction organisations and project-based procurement of works [17]. Size of company [18–20], age [21–24], gender [18–20, 25], education [19] and type of work [26–28] are also associated with occupational injury.

In the developed countries, several attempts have been made to investigate factors influencing safety performance on construction sites. However, the predisposing factors for occupational injury in the construction sites in Ethiopia are not well investigated. This study was, therefore, conducted to assess the magnitude of occupational injury and associated factors among construction workers in Gondar town, northwest Ethiopia.

## Methods

### Design and settings of the study

An institutional-based cross-sectional study design was conducted among construction workers in Gondar town. More than 20 construction sites were found in Gondar town as of April 2015 and the major sites are owned by eight different companies. Around 2586 workers were engaged in different working units in all sites. Workers who directly engaged in different working units were considered as study subjects and workers who were not engaged in construction works like administrative workers were excluded from the study.

### Sample size determination

The sample size was determined using single population proportion formula with the following assumptions: prevalence of occupational injury (p) = 38.7% [29], margin of error (w) = 4, 95% confidence interval (standard normal probability), and level of significance (α) = 4. Therefore, the sample size (n) was computed as $$ n=\frac{{\left({z}_{\raisebox{1ex}{$\alpha $}\!\left/ \!\raisebox{-1ex}{$2$}\right.}\right)}^2p\left(1-p\right)}{w^2}=\frac{(1.96)^20.387\ \left(1-0.387\right)}{0.04^2}=569 $$. The final sample was found to be 596 considering 5% non-response rate.

### Sampling technique and sampling procedure

More than 20 construction sites with more than 2586 workers are found in Gondar town as of April 2015. Eight construction sites were selected using simple random sampling technique. All workers who actively engaged in different sections were included and persons who had no exposure to occupational hazards like office workers were excluded from the study. Hence, the number of workers at each site varied; the sample size was proportionally allocated (Table 1). Finally, the study subjects from each site were selected by simple random sampling technique (using random number generator) using workers in the registration book as a sampling frame.

**Table 1:** Distribution of study participants in the eight construction sites in Gondar town, northwest Ethiopia, April 2015

Construction sites	Number of participants
Amhara Wuhawoch Drigit	303
Nigidu Kibrit	105
Unity Engineering	58
3 M Construction	53
Medrock Construction	27
Afrotsione Construction	22
Alemayew Ketema Construction	15
Aielmi Construction	13

### Description of study variables

#### Dependet variable

Occupational injury, the primary outcome variable of this study, is defined as any physical damage of the human body or tissue like laceration, cut, puncture, fracture, dislocation, amputation, electrocution, ear injury, and eye injury results from harmful contact between people and objects, substances, or other things in their surroundings.

#### Independet variables

##### Utilization of PPEs

Is defined as use of suitable protective equipment like hand glove, toetector/feet wear, respirator, face mask, reinforced cloth, goggle and helmet on duty.

##### Manual handling

Is defined as any load by physical effort incorporates lifting, pushing, pulling, putting down, caring and moving from the ground level and higher level.

##### Health and safety training

Is any formal or informal health and safety education provided for workers to create basic understanding of occupational health, workplace hazards, injury prevention and safety.

##### Attention to work

Is a physical and mental due attention or focus given for the work only.

##### Alcohol use

Male and female participants who drink more than six and five glasses or bottles or cans of any alcoholic beverage, such as beer, wine and Tela (local beverage) respectively on a regular work or weekend days, including the off-work hours were considered as drunker.

##### Hazard awareness

Is defined as an existed knowledge or understanding of occupational hazards.

Moreover, health and safety supervision, working hours per week, working section, and socio-demographic characteristics were other predictor variables considered in this study.

### Data collection tools and data collection procedures

Data were collected using a structured questionnaire and observational checklists. The data collection tools were adopted from other similar published studies [29–31] with simple modification to address contextual issues related to study setting and participants. The questionnaire consisted of socio-demographic information, work-related injury characteristics, work environment and ergonomic related information, and workers behavior-related information. The tools were pretested on workers who were not actually part of the study having similar characteristics with the study subjects in different town and necessary correction was done. Training was given for data collectors and supervisors on data collection procedures and data collection tools. The overall physical condition of the workers and the working condition were observed using checklists [32–35]. Working conditions and safety practices were the items included in the checklists. We immediately checked and corrected completeness of data before the collectors move to the next interview. Supervisors checked the completeness of all the filled questionnaires daily and 5% of the collected questionnaires were repeated. Furthermore, double data entry and software assisted data cleaning were employed.

### Data processing and analysis

Data were entered using EPI-INFO version 3.5.3 and exported to statistical package for social sciences (SPSS) version 20.0 for further analysis. Univariable binary logistic regression analysis was used to choose variables for the multivariable binary logistic regression analysis on the basis of *p*-value less than 0.2. In the multivariable binary logistic regression analysis, statistically significant variables were identified on the basis of AOR with 95% CI and *p* < 0.05. Model goodness-of-fit was checked by Hosmer-Lemeshow test. Multicollinearity was also checked.

## Results

### Socio-demographic characteristics

In this study, 566 construction workers participated. This gives 95% response rate. Of these, 295 (52.1%) were male. The mean age of the participants was 25.78 years with ±6.58 standard deviation. Three hundred fifty (61.8%) of the workers attended primary education. Three hundred ninety-nine (70.5%) of the study subjects had five and below years of work experience in the construction industry. About two-third, 358 (63.3%) of the construction workers were daily labourers (Table 2).

**Table 2:** Socio-demographic characteristics of construction workers (*n* = 566) in Gondar town, April 2015

Socio-demographic variables	Frequency	Percent
Sex		
Female	271	47.9
Male	295	52.1
Age		
14–29	550	97.2
> 30	16	2.8
Educational status		
Blow grade 8	350	61.8
9–12 grade	160	28.3
Diploma and above	56	9.9
Marital status		
Married	196	34.6
Separated	301	53.2
Single	69	12.2
Monthly income		
≤ 1000	367	64.8
> 1000	199	35.2
Working experience		
≤ 5 years	399	70.5
> 5 years	167	29.5
Occupational title		
Daily labors	358	63.3
Carpenter	74	13.1
Builders	47	8.3
Plaster	36	6.4
Driver/Operator1	32	5.6
Welders/electrician	19	3.4
Daily labors which involving the work (*n* = 358)		
Lifting and carrying stone and cement	164	45.8
Helping the painter	75	20.9
Helping the builders	45	12.6
Mason	40	11.2
Helping the carpenter	34	9.5

### Working condition and workers behaviour

Fifty-four (9.5%) of the respondents worked for more than 48 h per week. Four hundred fifty-seven (80.7%) of the respondents had not been regularly supervised at work and 483 (85.3%) of the study subjects had not ever taken safety and health training. Four hundred sixty-three (81.8%) of the study subjects responded that their job involved manual handling and 530 (93.6%) of the workers worked with vibrating hand tools (Table 3). Four hundred fifty (79.5%) of the respondents were not using PPEs while working. The most frequently reported reason for not using PPEs was shortage of devices, which accounts for 98.2%. The remaining 116 (20.5%) of workers used PPEs. Helmet (33.6%), glove (27.6%), overall (18.1%), boots/shoes (8.6%), earplug (6.9%), respirator (2.6%), and goggle (2.6%) were commonly reported PPEs. The result of this study also showed that 17 (3%), 318 (56.2%) and 16 (2.8%) of the study subjects were smoker, drunker, and chat chewer respectively.

**Table 3:** Working environment and ergonomic related factors among construction workers (*n* = 566) in Gondar town, April 2015

Work environment and ergonomic related factors	Frequency	percent
Hours per week		
≤ 48 h	512	90.5
> 48 h	54	9.5
OSHS safety supervision		
Yes	109	19.3
No	457	80.7
Safety training		
Yes	83	14.7
No	483	85.3
Manual handling		
Yes	463	81.8
No	103	18.2
Weight of objects manually handled (*n* = 463)		
Light (not greater than 5 Kg)	132	28.5
Medium (6–10 Kg)	58	12.5
Heavy (11–20 Kg)	54	11.7
Very heavy (> 20 k.g)	219	47.30
Time spend on manual handling /day(n = 463)		
< 2 h	21	4.5
2–4 h	76	16.5
> 4 h	366	79
Concentration at the work		
Yes	492	86.9
No	74	13.1
Working with vibrating hand tools		
Yes	530	93.6
No	36	6.4
Hazard awareness		
Yes	497	87.2
No	69	12.2

### Magnitude of occupational injury

**Fig. 1:**
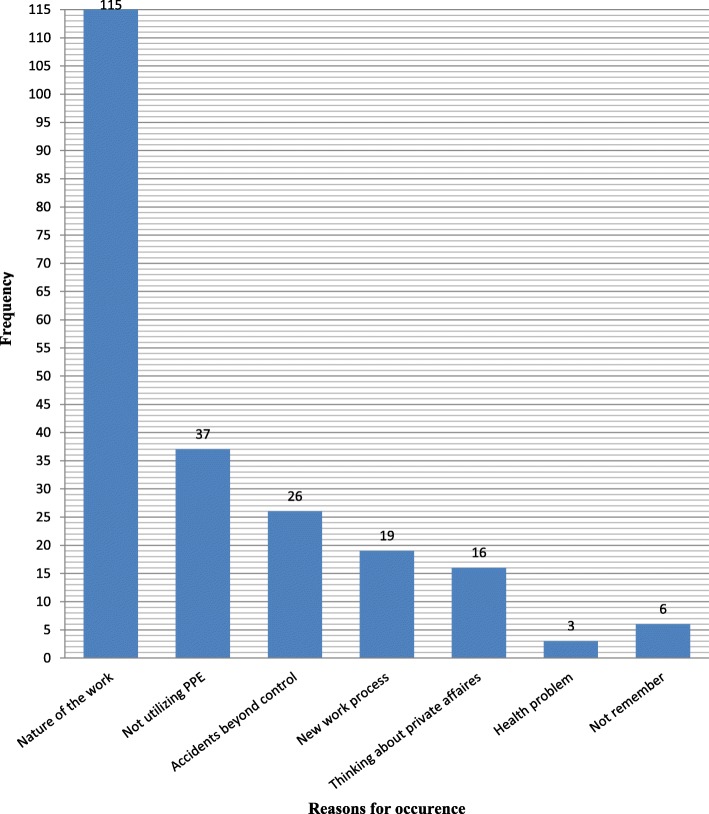
Reported reasons for occurrence of work-related injury by construction workers in Gondar town, April 2015

Out of 566 construction workers who directly engaged at different working units, 221 were injured in the last 3 months. Therefore, the overall prevalence of work-related injury was found to be 39% (95% CI = 35.0–43.1%). Of the injured workers, 56 (25.34%) experienced work-related injury more than once (Table 4). Abrasion or laceration was the leading type of injury, which accounts for 108 (48.75%). The commonest causes of injury were found to be cut by sharp objects, 62 (28.05%) and fall to ground level, 52 (23.54%) (Table 5). The two most reported reasons for injury were the nature of the work (52.03%) and not using PPEs (16.74%) (Fig. 1). Eighty-four (38%) of injury cases were managed in the construction sites using first aid services whereas, 111 (50.2%) and 25 (13.3%) of the injured workers respectively reported as they visited health institution and as they used traditional medicine to manage the injury.

**Table 4:** Work-related injury among construction workers in Gondar town (*n* = 566), Ethiopia, April 2015

Occurrence of injury	Frequency	percent
Injury in the last 3 months		
Yes	221	39.0
No	345	61.0
Number of occurrence		
Once	165	76.7
More than once	56	25.3
Injury in the last 2 weeks		
Yes	77	13.6
No	489	86.4
Number of occurrence		
Once	68	88.3
More than once	9	11.7

**Table 5:** Type and causes of injury and body parts affected among workers (*n* = 221) in construction enterprise, Gondar town, Ethiopia, April 2015

Variables	Frequency	percent (%
Types of injury		
Abrasion /laceration	108	48.9
Puncture	36	16.3
Dislocation	20	9.0
Eye injury	17	7.7
Cut	14	6.3
Fracture	13	5.9
Back pain	8	3.6
Electrocution	2	0.9
Amputation	1	0.5
Causes of injury		
Cut by sharp objects	62	28.1
Falls of the ground level	52	23.5
Falling from the height	43	19.5
Hit by falling objective	17	7.6
Being struck machine	14	6.3
Over exertion during lifting	18	8.1
struck by moving machine	9	4.1
Contact electric line	5	2.3
Others	7	3.2
Parts of the body affected		
Hand	88	39.8
Toes	45	20.4
Eye	29	13.0
Leg figures	13	5.8
Head	9	4.0
Back	9	4.0
Upper leg	6	2.7
Lower leg	6	2.7
Chest	4	1.8
Upper arm	4	1.8
Knee	3	1.3
Lower arm	1	0.5
Ear	1	0.5
Others	3	0.1

### Factors associated with occupational injury

Sex, marital status, service year, safety training, attention to work, work with vibrating hand tools, hazard awareness, and alcohol consumption were selected for the multivariable analysis. As clearly indicated in Table 6, marital status, service year, attention to work, work with vibrating hand tools, awareness about occupational hazards and alcohol consumption were statistically associated with the occurrence of occupational injury in the final model. The occurrence of occupational injury among single construction workers was 50% to be lower compared with married workers [AOR = 0.50, 95% CI = 0.25, 0.97]. The likelihood of injury occurrence among workers who worked for more than 5 years was 2.79 times to be higher compared with workers who worked for five and below years [AOR = 2.79, 95% CI = 1.72, 4.53]. Those construction workers who had no attention to work were 2.65 times to be injured compared with their counterparts [AOR = 2.65, 95% CI = 1.33, 5.29]. Workers who worked with vibrating hand tools were 3.23 times to be injured [AOR = 3.23, 95% CI = 1.19, 8.76]. Workers who had no awareness about occupational hazards were 4.66 times more likely to be injured [AOR = 4.66, 95% CI = 1.99, 10.87]. The occurrence of injury was 3.16 times to be higher among construction workers who are drinkers [AOR = 3.16, 95% CI = 2.09, 4.79].

**Table 6:** Factors associated with occupational injury (*n* = 566) in Gondar town construction enterprise, April 2015

Predictor variables	Occupational injury		COR (95% CI)	AOR(95% CI)
	Yes	No		
Sex				
Female	126	145	1.00	1.00
Male	95	200	1.83 (1.31, 2.60)	1.43 (0.95, 2.17)
Marital status				
Married	85	111	1.00	1.00
Separated	97	204	1.61 (1.11, 2.33)	1.32 (0.84, 2.08)
Single	39	30	0.60 (0.34, 1.05)	0.50 (0.25, 0.97)*
Service year				
< 5 years	188	211	1.00	1.00
> 5 years	33	134	3.61 (3.35, 5.54)	2.79 (1.72, 4.53) ***
Safety training				
Yes	42	41	1.00	1.00
No	179	304	1.74 (1.09, 2.78)	1.46 (0.84, 2.53)
Concentration at work				
Yes	208	284	1.00	1.00
No	13	61	3.43 (1.84, 6.42)	2.65 (1.33, 5.29)**
Work with vibrating tools				
Yes	215	315	1.00	1.00
No	6	30	3.41 (1.39, 8.34)	3.23 (1.19, 8.76)*
Hazard awareness				
Yes	214	283	1.00	1.00
No	7	62	6.69 (3.00, 14.92)	4.66 (1.99, 10.87)***
Alcohol consumption				
Yes	160	158	1.00	1.00
No	61	187	3.10 (2.15, 4.46)	3.16 (2.09, 4.79)***

Statistically significant variables at *p* < 0.05 | ** Statistically significant variables at *p* < 0.01| *** Statistically significant variables at *p* < 0.001| The result of Hosmer and Lemshow test was > 0 .670 | VIF = 4.5

## Discussion

The overall three-month prevalence of occupational injury among construction workers in Gondar town was 39% (95% CI = 35.0–43.1%). The prevalence reported in this study is the same with findings of studies in Gondar city, 38.7% [30]; Maraki campus, University of Gondar, 38.7% [29]; Southeastern Ethiopia, 41.4% [36]; southwestern Ethiopia, 39.2% [37]; Addis Ababa, 38.3% [35]; and Nigeria, 39.25% [38]. The result of this study is lower than the results of other studies in Addis Ababa, 84.7% [31] and 67.7% [39], Iran, 79.8% [40]; Tamil Nadu, 44.3% [41]; and Egypt, 46.2% [42]. The result of this study is also higher than the report of other studies in Gondar, 15% [43]; Uganda, 32.4% [44]; and China 34.82% [45]. This difference might be due to differences in study settings, working conditions, level of accident prevention strategies, and socio-cultural and regulatory factors. In this study, the prevalence of occupational injury is high may be due to no regular workplace supervision, poor PPEs utilization, use of manually handled vibrating construction materials, and long working hours per week.

In this study, a significant proportion of young workers engaged in the construction industry. Young workers are at higher risk of occupational injury than older age groups. Young workers face higher occupational injury risks related to their higher vulnerability. Some known contributors to youth workplace injury include potential lack of specific job training. Many youth are not aware of their legal rights and are thus ill-equipped to identify potential hazards and request training to appropriately manage these hazards. Youth may also feel intimidated in the workplace. They may feel powerless to change their working conditions, or too shy to voice their concerns if they are new in their working environment [46–49].

In this study, the occurrence of occupational injury among single construction worker was 50% lower compared with married workers. The finding of this study is supported by another similar study [50]. This may be due to married workers may engage in other works without taking adequate rest. Stress and fatigue can be higher among married workers than single ones because of higher responsibilities in life. It may be led to more unsafe acts resulting in accident [50–52].

This study depicted that workers who reported longer working years had greater chance to occupational injury. This might be explained that accidents usually occur to workers who could still have had a long working career. Those engaged in routine activities for a long period with poor working environment may sustain job dissatisfaction; the work is insecure so that workers stayed for a long period in this insecure job had an increased vulnerability of different injury [35, 53–55].

The current study identified that due attention to work was associated with the occurrence of occupational injury. The odds of injury among construction workers who had no attention to work was higher than workers who had. This might be due to the fact that those workers who had no attention to work did not comply with standard work procedures, safety precautions including proper use of PPEs. In addition, workers who had no attention to work would also create hazards to their co-workers. There is some evidence to suggest that there is a link between accidents and distractibility, poor attention and mental error [56–58].

This study revealed that workers who used vibrating hand tools were more likely to be injured compared with their counterparts. This may be due to the type of hand tools in which construction workers used to operate their work is associated with their physical health. Physical work demands like vibration and heavy lifting aggravating the occurrence of injury. Vibrating tools can cause loss of muscle strength and reduced grip force due to incomplete muscle contraction [59].

This study found that occupational injury was significantly associated with awareness about occupational hazards. The odds of occupational injury among construction workers who had no awareness about occupational hazards was more likely to be higher. The association of awareness and injury can be justified that workers who were not aware about workplace hazards, prevention of injury and other safety measures may do work with wrong procedures and may not also comply with workplace safety strategies [39, 60–62].

This study indicated that the occurrence of occupational injury was associated with alcohol consumption. Construction workers who took alcohol were more likely to be injured compared with their counterparts. This finding is consistent with the findings of other studies [55, 63, 64]. This may be due to the fact that alcohol can impair judgmental and psychomotor skills. Alcohol took before work begins can cause effects such as fatigue and hangovers. Alcoholic workers may be more likely to be engaged in other behaviors that increase the risk of injury [65–67].

### Limitation of the study

Workers who were absent from work due to illness were not included in this study. Therefore, the results of the study might be affected by healthy workers effect. Authors tried to check whether the absenteeism was due to occupational injury or not to minimize healthy workers effect.

## Conclusion

High prevalence of occupational injury was reported in the study area. Cut and fall were the leading causes of injury. Marital status, service year, attention to work, use of vibrating tools, awareness about occupational hazards, and drinking alcohol were identified as factors associated with occupational injury. Therefore, health and safety trainings have to be taken place to aware workers about occupational injury and safety issues. Regular workplace supervision and provision of PPEs are also needed to prevent occupational injury. The findings of this study are useful to design and implement injury prevention strategies in the country. The study also contributes to the current literature as health and safety information is limited, especially in developing countries.

## Data Availability

Data will be made available upon requesting the primary author.

## Abbreviations

AOR: Adjusted Odds Ratio
CI: Confidence Interval
COR: Crude Odd Ratio
PPEs: Personal Protective Equipment
SPSS: Statistical Package for Social Science
VIF: Variance inflation factor
